# Evolutionary Trends in the Adoption, Adaptation, and Abandonment of Mobile Health Technologies: Viewpoint Based on 25 Years of Research

**DOI:** 10.2196/62790

**Published:** 2024-09-27

**Authors:** Jennifer Portz, Susan Moore, Sheana Bull

**Affiliations:** 1 Division of General Internal Medicine School of Medicine University of Colorado Aurora, CO United States; 2 mHealth Impact Lab Colorado School of Public Health University of Colorado Aurora, CO United States

**Keywords:** technology adoption, mobile health, SMS text messaging, mobile apps, wearables, social media

## Abstract

Over the past quarter-century, mobile health (mHealth) technologies have experienced significant changes in adoption rates, adaptation strategies, and instances of abandonment. Understanding the underlying factors driving these trends is essential for optimizing the design, implementation, and sustainability of interventions using these technologies. The evolution of mHealth adoption has followed a progressive trajectory, starting with cautious exploration and later accelerating due to technological advancements, increased smartphone penetration, and growing acceptance of digital health solutions by both health care providers and patients. However, alongside widespread adoption, challenges related to usability, interoperability, privacy concerns, and socioeconomic disparities have emerged, necessitating ongoing adaptation efforts. While many mHealth initiatives have successfully adapted to address these challenges, technology abandonment remains common, often due to unsustainable business models, inadequate user engagement, and insufficient evidence of effectiveness. This paper utilizes the Nonadoption, Abandonment, Scale-Up, Spread, and Sustainability (NASSS) framework to examine the interplay between the academic and industry sectors in patterns of adoption, adaptation, and abandonment, using 3 major mHealth innovations as examples: health-related SMS text messaging, mobile apps and wearables, and social media for health communication. Health SMS text messaging has demonstrated significant potential as a tool for health promotion, disease management, and patient engagement. The proliferation of mobile apps and devices has facilitated a shift from in-person and in-clinic practices to mobile- and wearable-centric solutions, encompassing everything from simple activity trackers to advanced health monitoring devices. Social media, initially characterized by basic text-based interactions in chat rooms and online forums, underwent a paradigm shift with the emergence of platforms such as MySpace and Facebook. This transition ushered in an era of mass communication through social media. The rise of microblogging and visually focused platforms such as Twitter(now X), Instagram, Snapchat, and TikTok, along with the integration of live streaming and augmented reality features, exemplifies the ongoing innovation within the social media landscape. Over the past 25 years, there have been remarkable strides in the adoption and adaptation of mHealth technologies, driven by technological innovation and a growing recognition of their potential to revolutionize health care delivery. Each mobile technology uniquely enhances public health and health care by catering to different user needs. SMS text messaging offers wide accessibility and proven effectiveness, while mobile apps and wearables provide comprehensive functionalities for more in-depth health management. Social media platforms amplify these efforts with their vast reach and community-building potential, making it essential to select the right tool for specific health interventions to maximize impact and engagement. Nevertheless, continued efforts are needed to address persistent challenges and mitigate instances of abandonment, ensuring that mHealth interventions reach their full potential in improving health outcomes and advancing equitable access to care.

## Introduction

mHealth, short for mobile health, represents a transformative approach to health care delivery by harnessing the power of mobile devices such as smartphones, tablets, and wearables to enhance communication, collect health data, and monitor well-being [1]. This technology has the potential to revolutionize health care services by offering personalized care, improving access, reducing costs, and empowering patients in managing their health [2]. With the proliferation of smartphones, even among traditionally underserved populations, and the widespread availability of internet access, the stage is set for the broad adoption of mHealth solutions [3,4]. However, the full potential of mHealth has yet to be realized [5].

At the heart of mHealth’s potential is the theory of diffusion of innovations, which explains how new ideas, technologies, and practices spread within a society or organization [6]. In the context of mHealth, this diffusion process involves the adoption of mHealth technologies by various stakeholders, including health care providers, patients, and policy makers. Several factors influence the diffusion of mHealth innovations. First, the perceived benefits of these technologies play a crucial role. Innovations that promise to improve health care access, efficiency, and outcomes are more likely to be embraced by users. Second, the ease of use and accessibility of mHealth solutions are critical determinants of their adoption. Particularly among marginalized populations, including racial and ethnic minorities, individuals with low socioeconomic status, older adults, and those with disabilities, ensuring that mHealth tools are user-friendly and accessible is essential for equitable adoption.

Despite the promise of mHealth, challenges remain in achieving widespread adoption. Concerns regarding usability, privacy, data security, and the potential replacement of in-person care with virtual interactions have led to hesitancy among both patients and providers [7]. Additionally, interoperability issues between mHealth apps and electronic health record systems pose challenges for seamless integration into clinical workflows, potentially increasing provider burden [8]. Furthermore, the complexity of reimbursement and regulatory mechanisms presents obstacles to the large-scale implementation of mHealth solutions [9]. Without clear guidelines on reimbursement for mHealth services and adherence to regulatory requirements, health care organizations may hesitate to invest in these technologies. While mHealth holds tremendous promise for revolutionizing health care delivery, its widespread adoption depends on addressing implementation challenges and ensuring that innovations are accessible, user-friendly, and aligned with regulatory and reimbursement frameworks. By leveraging the principles of diffusion of innovations and addressing barriers to adoption, mHealth has the potential to transform health care and public health, improving outcomes for individuals, families, and communities.

In this viewpoint, we use the innovation-diffusion Nonadoption, Abandonment, Scale-Up, Spread, and Sustainability (NASSS) framework [10] to examine the dynamic relationship between the academic and industry sectors concerning adoption, adaptation, and abandonment patterns of 3 prominent mHealth innovations: health-related SMS text messaging, mobile apps and wearables for chronic disease management, and the use of social media for health communication. By utilizing these innovations as case studies, the paper will explore their potential in health promotion, disease management, and patient engagement. This viewpoint aims to provide insights into the ongoing trends in innovation and adoption within the mHealth landscape.

## Innovation and Diffusion

To consolidate various innovation diffusion theories, the NASSS framework emerged. It aims to guide the development of novel technologies that are conducive to widespread scalability while also identifying emerging technologies with limited potential [10]. NASSS represents a comprehensive, health care–specific paradigm that underscores the interplay among individual factors (micro), organizational dynamics (meso), and overarching policy frameworks (macro) in facilitating or hindering technology adoption. By harnessing the analytical power of NASSS, insights into technological shortcomings can be gathered, enhancing preparedness for implementation. NASSS notably delineates several key domains: (1) the nature of the health condition(s); (2) the technological features and types of data collected; (3) the value proposition for patients, industry, and clinical stakeholders; (4) the composite adopter system, including health care professionals, patients, and informal caregivers; (5) organizational structures such as capacity and readiness to adopt; (6) broader institutional, societal, and cultural contexts; and (7) the dynamic interactions and reciprocal adaptations among these domains over time. While barriers to adoption and adaptation are categorized as simple, complicated, or complex, technology solutions addressing complex challenges that span multiple domains rarely achieve mainstream acceptance. A summary of our viewpoint is provided in Table 1.

**Table 1:** Viewpoint summary by NASSS^a^ dimension.

NASSS dimension	SMS text messaging	Mobile health and wearables	Social media
Condition	Effectiveness in promoting healthy behaviors, smoking cessation, weight management, and chronic condition self-management. SMS text messaging was invented in 1984; widely used by 1999; popular by 2000; and now an average of 32 texts/day. Limitations in addressing cultural differences and sociocultural context.	Mobile apps offer health tracking, personalized coaching, educational resources, behavioral interventions, and remote monitoring. Wearables monitor metrics, such as physical activity, sleep, heart rate, and nutrition, integrated into mobile apps. The early 2000s saw a rise in health-related mobile apps with basic tracking functions. Now over 350,000 health apps are available. Limitations in addressing cultural differences and sociocultural context.	Technology for communication and information sharing among virtual communities. Early systems such as bulletin board systems, Usenet^b^, and Internet Relay Chat (1980s); web-based communities such as webrings and GeoCities (1990s); first social media platform: Six Degrees (1997). Now over 5 billion users worldwide, representing 62.3% of the global population. Limitations in addressing cultural differences and sociocultural context.
Technology	SMS text messaging is highly accessible, easy to implement, and scalable in health programs. Automated systems can be developed in-house or through third-party vendors. Difficulty in verifying SMS text message comprehension and engagement.	Mobile apps require diverse technologies and skills, developed for operating systems such as iOS and Android. Security measures are crucial for protecting user data, and regular updates and maintenance are vital for app reliability and user experience.	Varies in communication modalities (text, image, audio, and video). Central to social media; includes replies, shares, likes, and other engagement types, often permission-based.
Value proposition	Cost-effective promotion: SMS text messaging programs are low-cost. Health care systems can reach thousands with minimal cost per SMS text message. Effectiveness: Texting interventions can be effective, but impact is typically small to medium; scaling is necessary for significant impact.	User empowerment: Apps and wearables offer personalized tools for health management, fostering autonomy, self-efficacy, and empowerment. Bidirectional engagement: Personalization, gamification, and social support enhance user engagement and motivation.	Extensive reach: Ability to tailor messages to specific communities and transmit information rapidly. Cost-effective promotion: Low-cost messaging with potential for viral spread. Data analysis: Identifies emerging health concerns, traces outbreaks, and tracks disease spread.
Adopters	Health care providers: Providers endorse SMS text messaging but are reluctant to engage directly due to concerns about boundaries and being overwhelmed with SMS text messages. Automated systems allow delegation, limiting direct provider-patient communication. Challenges: Older adults and the visually impaired engage less with texting. Limited use of literacy-sensitive and multilingual SMS text messaging. Limited evidence of SMS text messaging’s impact on caregivers.	Widespread use: Mobile apps and wearables are used across diverse populations, but adoption varies due to clinical, demographic, and technological factors. Challenges: Challenges include technology literacy, cost, security concerns, accessibility for older adults and low-income communities, and limited internet connectivity in rural areas.	Widespread use: Nearly ubiquitous among technology users; billions on platforms such as Facebook. Challenges: Adoption varies by platform and demographics; health campaigns should tailor messages to specific populations.
Organization	Organizations use SMS text messaging for appointment reminders and medication refills. Regulatory barriers limit the widespread use of prevention and self-management programs. Barriers to making texting a standard of care include resource constraints, motivation, and regulatory issues.	Facilitated by seamless integration into workflows. Engagement with user feedback promotes effective adoption in clinical care settings. Challenges including organizational resistance, limited resources, data privacy concerns, and interoperability issues hinder adoption.	Engagement is affected by policies on who can post and what content is shareable. Involves content experts and communications experts; slow approval processes can hinder timely responses. Requires understanding of cultural contexts and platform-specific norms for successful strategies.
Wider system	Regulatory challenges, security and consent, and potential for sociocultural tailoring of SMS text messages, yet set up is costly and complex.	Regulatory challenges, interoperability with existing systems, digital divide, and resistance from health care providers.	Approachability and trust of social media by health care systems; navigating health policies alongside social media policies is complex; and the importance of maintaining accountability, combating misinformation, and addressing harassment in social media.
Embedding and adaptation over time	Interest in AI^c^-enabled chatbots as a sophisticated iteration of SMS text messaging. AI chatbots offer interactive and personalized communication but face similar implementation challenges as current texting programs.	Requires collaboration, resource allocation, long-term funding, and viable business models to expand initiatives and reach broader populations effectively.	Health communication strategies must consider potential negative impacts to avoid unintended consequences.

^a^NASSS: Nonadoption, Abandonment, Scale-Up, Spread, and Sustainability.

^b^Usenet: User’s Network.

^c^AI: artificial intelligence.

## SMS Text Messaging

### Condition

SMS text messaging can be used to promote healthy behaviors and address various conditions and comorbidities in a culturally responsive manner. Although SMS text messaging was invented in 1984, it was not widely adopted until 1999, when users began texting across different phone carrier networks. By 2000, SMS text messaging had become popular, with SMS text message volumes averaging around 35 texts per person per month in the United States [11]. The earliest evidence of SMS text messaging’s efficacy for health emerged in the first decade of the 21st century, with publications demonstrating its potential impact on self-management of type 1 diabetes, drinking behaviors, and medical appointment adherence [12-14]. The popularity of SMS text messaging in daily life has continued to accelerate, with people now receiving an average of 32 texts per day (range 16-128 texts per day). A lower number of SMS text messages is typically sent among older individuals, while younger people tend to receive higher volumes [15]. With this increased popularity, there has been a concomitant rise in studies exploring how SMS text messages can impact health behaviors, prevention, and the self-management of both chronic and infectious conditions. We now have a robust body of literature, including meta-analyses, demonstrating that SMS text messaging can be effective in facilitating healthy behaviors [16-22]. Numerous meta-analyses have focused on smoking cessation, weight management, medication and appointment adherence [23-28], and self-management of chronic conditions [29-32]. While this literature is extensive, researchers have consistently acknowledged limitations in using SMS text messaging to create robust programs that incorporate cultural differences and the sociocultural context of patients and populations. However, there are notable exceptions that demonstrate the feasibility of integrating cultural context into SMS text messaging programs. Examples include a smoking cessation program for Māori in New Zealand [33] and another focused on promoting healthy behaviors in low- and middle-income country settings [34].

### Technology

SMS text messaging is a highly accessible and easy-to-implement technology, which enhances its potential for scalability and widespread dissemination in health care. It does not require complex technological architecture. Automated programs can be developed in-house or utilized through third-party vendors contracted with health care delivery systems. However, it can be challenging to obtain clear evidence that people are reading and understanding SMS text messages. Automated systems can document that SMS text messages are sent and received, but they cannot confirm that they have been read or that people comprehend them [35]. This is particularly true for unidirectional SMS text messages, where people cannot respond to an SMS text message or their responses are not cataloged. Given that smartphone ownership with texting capability is nearly universal in the United States [36], and texting behaviors are widespread among smartphone users [15], there is no additional knowledge required for people to access and engage with SMS text messages. However, we note that there are barriers for some people in accessing and using SMS text messaging, which we will discuss further below.

### Value Proposition

The value proposition of SMS text messaging can only be realized when programs are scaled. Developers stand to gain significantly from building SMS text messaging programs due to the relative simplicity of coding such systems, which results in relatively low production costs. However, this low cost also means that a return on their development investment will be achieved only when thousands of users access and utilize these systems [37]. Health care delivery systems also face very low costs in implementing texting programs; SMS text messages are sent and received for just a few cents and can easily reach thousands or even tens of thousands of patients through texting campaigns. While SMS text messaging interventions have been shown to be effective, their effects are generally small and occasionally moderate [38]. Texting campaigns can only be impactful when scaled to thousands of users. The widespread use and growing popularity of texting as a daily communication medium mean that it may become increasingly difficult for health care delivery systems to send SMS text messages that resonate or compete for attention against the dozens, or even hundreds, of SMS text messages individuals receive each day [15].

### Adopters

While people have adopted SMS text messaging with enthusiasm, challenges with reach and engagement remain. Although health care providers may support the use of SMS text messaging from their clinics and care delivery organizations to patients, they often show reluctance or resistance to directly engage with patients via SMS text messages. Providers cite concerns such as being inundated with SMS text messages from patients and having professional boundaries violated, such as being expected to respond to SMS text messages at night and on weekends [39]. The widespread use of automated systems to send SMS text messages to patients across entire health care systems allows providers to delegate texting tasks to other staff, keeping their involvement limited to a more tangential role. While this approach may effectively manage patient-provider communication, it also means that providers might not see the SMS text messages patients are sending, potentially missing opportunities to better understand the daily challenges patients face with prevention and self-management behaviors.

While texting is highly popular and accessible at the population level, some groups, such as older adults and individuals with visual impairments, engage less with SMS text messages due to accessibility concerns, such as font size or limited access to text-to-speech features [40]. Ongoing critiques of SMS text messaging literacy suggest that greater accessibility could be achieved by paying more attention to literacy levels [41]. Additionally, there is a lack of widespread use of multilingual SMS text messaging, which could improve accessibility for non-English speakers [42]. Much of the literature on SMS text messaging in health has focused on the impacts of interventions at the individual level. There is less evidence showing the impact on caregivers [43], which represents a missed opportunity given that caregivers could help older adults or individuals with impairments access and improve care with support from SMS text messages.

### Organization

While organizations have adopted SMS text messaging for certain tasks, there is a lack of consistent adoption and implementation of prevention and self-management programs across patient lifespans. There are multiple examples of organizations using SMS text messaging to remind patients to schedule and keep appointments and to pick up or refill prescribed medications [23]. While the capacity to innovate may have driven earlier adoption of SMS text messaging systems, organizations still face challenges in widespread use for diverse prevention and self-management programs due to regulatory barriers, which we discuss below. The ability for organizations to develop texting programs in-house may be less relevant given the widespread availability of texting platforms from third-party vendors. Organizations can make texting campaigns attractive and valuable when they focus on key objectives, such as increasing childhood vaccine coverage [44] or screening for colorectal cancer [45]. However, the widespread institutionalization of systems integrating texting for primary prevention, health behavior, and self-management as a standard of care remains elusive. It is unclear whether this is due to resource constraints, motivation, willingness to change, or other unknown factors.

### Wider System

Understanding the context of texting within the framework of policies and regulations is crucial. While individual organizations and health care delivery systems might be able to implement texting as a standard of care for specific use cases (eg, to encourage flu vaccine uptake), several regulatory requirements limit its widespread institutionalization as envisioned. The Health Insurance Portability and Accountability Act (HIPAA) requirements for confidentiality and privacy restrict what can be shared in outbound patient messages and necessitate security measures that may complicate access via cell phones. Additionally, the security protocols in electronic health records have created firewalls that make direct texting to patients difficult without complex integrations with patient portals. Organizational adherence to HIPAA is influenced by a recent ruling from the Federal Communications Commission, which requires organizations sending SMS text messages to obtain explicit permission from individuals. This adds an additional layer of effort for organizations to enroll patients in SMS text messaging programs and may bias program participation by excluding those who cannot be reached or choose not to opt-in [46]. While automated texting has the potential to enhance attention to sociocultural variability by allowing individual tailoring to patients, setting up the necessary infrastructure and programming can be daunting and initially costly for health care delivery systems. We will elaborate on this topic in the following paragraphs.

### Embedding and Adaptation Over Time

Embedding and adapting texting over time requires aligning incentives for health care organizations. As discussed in the preceding paragraphs, there is substantial evidence that SMS text messaging can improve health behaviors and outcomes. Given that this evidence has been available for nearly 2 decades, what prevents texting from being embedded in health care delivery systems as a standard of care to support healthy behaviors and self-management across the lifespan? The regulatory challenges mentioned above, the complexity of integrating texting with electronic health records, and concerns about overwhelming patients and providers with texting fatigue are key reasons for the lack of universal texting integrations despite their potential. Additionally, there is insufficient alignment of incentives for organizations to use texting alongside other interventions. Numerous incentives within value-based care frameworks can be enhanced through texting interventions, such as improving colorectal, breast, and cervical cancer screening; screening for diabetes, hypertension, and cholesterol; and increasing flu, COVID-19, and childhood vaccine coverage.

While our assessment here has focused specifically on texting, it is important to note the growing interest in exploring the potential of more advanced SMS text messaging technologies, such as artificially intelligent (AI) chatbots [47-50]. AI-enabled chatbots advance automated communication beyond unidirectional texts and bidirectional texts with fixed response options (eg, text “1” if you need information on smoking cessation). Unlike these methods, AI-enabled chatbots allow users to initiate and direct their questions, providing a more personalized interaction. These systems interpret and classify queries using advanced language models—large neural networks trained on extensive text corpora and fine-tuned for accurate classification in domain-specific text processing. They can engage in conversations with patients on topics of their choice. However, embedding these advanced systems into health care delivery systems will face the same challenges as current texting programs.

## Mobile Apps and Wearables

### Condition

Mobile apps and wearables offer versatile functionalities for promoting healthy behavior and managing various health conditions and comorbidities. The earliest health-related mobile apps emerged in the early 2000s with the advent of smartphones and the integration of basic health features [51]. These early apps primarily focused on basic health tracking, such as recording physical activity, monitoring nutrition intake, and tracking vital signs (eg, heart rate and blood pressure) [52]. Modern mobile apps typically offer a broader range of functionalities beyond SMS text messaging. They often include features for health tracking, personalized coaching, access to educational resources, behavioral interventions, and remote health monitoring, among others. Combined with wearables equipped with sensor technology, these tools enable comprehensive health tracking and monitoring of metrics such as physical activity, sleep patterns, heart rate, and nutrition intake. This empowers individuals to gain insights into their health status and make informed decisions [53,54]. There are over 350,000 health-related mobile apps available, with approximately 250 new apps released daily, accounting for 47% of all apps [55,56]. Health-related app use varies by individual characteristics, but an estimated 70% of adults in the United States have used an app to track their health [57]. Early wearables, such as pedometers and heart rate monitors, primarily targeted individuals interested in exercise and fitness. By the mid-2010s, the rise of smartwatches and fitness trackers led to the integration of wearable data directly into mobile apps [58,59].

The scientific evaluation of effectiveness and safety has not kept pace with the rapid expansion of the commercial health-related app market, creating a disconnect between scientific literature and available apps [50,60]. Mobile apps developed and tested in clinical settings are rarely widely available in app stores, while commercially developed apps often lack high-quality effectiveness testing [61-63]. Despite the growing interest and proliferation of apps across popular platforms, as well as increased research focus on their evaluation, their influence on outcomes related to chronic diseases remains uncertain [64,65]. Systematic reviews and meta-analyses have produced varied findings regarding the impact of apps on clinical outcomes, self-management, and behavior change. This inconsistency raises concerns about the ability of scientific research to keep pace with rapid technological advancements. Many trials suffer from biases, such as high attrition rates and incomplete outcome data, and the effects on health outcomes and participant engagement often decline over time in longer-duration studies [66]. Existing evidence suggests that while mobile apps might offer some benefit compared with having no intervention at all, there is limited high-quality evidence demonstrating their effectiveness in enhancing health outcomes. This contrasts with lower-tech alternatives, such as SMS text messaging, which has a more robust evidence base. Similarly, wearables are increasingly used for various conditions and health topics [67]. They are reliable for measuring steps and heart rate but may be less accurate for capturing other sensor data, such as energy expenditure [68]. The impact of wearables on health outcomes such as exercise, weight, and biomarkers in populations with chronic conditions is also mixed [69].

### Technology

Developing a mobile app requires a diverse set of technologies, tools, and skills to create a functional, secure, and user-friendly app that meets the needs and expectations of its target audience [70]. Mobile apps are built on operating systems such as iOS by Apple Inc. and Android by Google (Alphabet Inc.). While these systems have similarities, they require different development kits and tools. Developers choose between them based on factors such as target audience, market share, and monetization models, which can impact an app’s success. There are 4 major development approaches: native, cross-platform native, hybrid, and progressive web apps, each with its own advantages and disadvantages [71]. Native apps are platform-specific and written in languages provided by the platform. Cross-platform apps, by contrast, compile into native code. Hybrid apps use web technologies bundled into app installation packages, while progressive web apps offer an app-like experience through browsers. Developers may prefer hybrid or cross-platform approaches due to the specialized skill set required for native development. These approaches utilize web technologies and frameworks within containers to mimic native apps. Backend development involves creating and managing services that support the mobile front end, known as an application programming interface (API). Cloud-based backend services are increasingly popular compared with on-site solutions, as they allow developers to focus on app features without worrying about additional infrastructure. The mobile front end, which is the user-facing aspect of the app, includes the visual and interactive elements, typically residing on devices or accessed through browsers. Development involves various team members, including designers and developers. Front ends interact with backend services via APIs, accessing data and updating backend systems. APIs facilitate communication between components and often integrate third-party functionalities, such as payment processing and social media integration, which enhances the user experience and enables cross-platform compatibility [72].

mHealth apps generate a wide range of data types to help users monitor and manage their health [73]. These include biometric data, such as heart rate and blood pressure; physical activity data, such as steps and calories burned; sleep data for tracking sleep patterns and quality; and nutrition data for logging food intake and monitoring dietary habits. Additionally, medication adherence apps record medication schedules and doses [74], symptom tracking apps allow users to monitor health indicators and communicate with health care providers [75], and health record apps provide easy access to personal electronic health records [76]. Developers build backend services or leverage databases from third-party providers, which are typically cloud-based but can also be on-site, to improve efficiency and productivity. Security measures safeguard user data through encryption techniques and secure authentication methods. Efficient database management dictates how an app stores, retrieves, and manages data, ensuring seamless user experiences and system reliability. Regular updates and maintenance are essential for sustaining app performance and delivering a reliable user experience amid evolving user needs and technological advancements.

### Value Proposition

The health-related mobile app and wearable market features a wide range of developers, from established health care organizations and technology companies to start-ups and independent developers. Monetization models include free apps with in-app purchases, subscription-based models, and paid apps. However, the majority of apps and wearables are currently not reimbursed by insurance or Medicare [77,78]. The market is highly competitive, with a continuous influx of new apps and features. The value of mobile apps and wearables for patients and consumers lies in their ability to empower individuals by providing personalized resources and tools for effective health management. These technologies are generally well-perceived by users, offering convenient access to health-related information and services and allowing individuals to monitor their health [79]. Personalization and tailoring, along with features such as gamification and social support, enhance engagement and motivation, encouraging users to adopt healthier habits and achieve their goals. Ultimately, mHealth apps and wearables empower individuals to actively participate in their health care, fostering autonomy, self-efficacy, and empowerment in managing their well-being.

### Adopters

People use mobile apps and wearables across different ages, genders, races, ethnicities, and health systems [36]. However, several factors contribute to the nonadoption of mHealth apps. Usage and access vary by clinical and demographic factors, with challenges including technology literacy, interest, cost, and security concerns [7]. Older adults and individuals with sensory impairments may have difficulty navigating touch interfaces or small screens, while low-income communities face obstacles related to the affordability of smartphones and data plans [80,81]. Additionally, rural populations may struggle with limited internet connectivity, non-English speakers may find comprehension challenging, and individuals with limited digital literacy skills or access to technical support may have difficulties effectively using mobile apps. These factors impact adoption rates among diverse user populations.

In addition to adoption challenges, barriers include insufficient early engagement with users to ensure successful app installation, a lack of direct communication with health care professionals, and limited interactive features. Concerns about technical issues and data security also deter sustained engagement. App usage initiation often depended on the ease of downloading and the individual’s motivation. Factors influencing engagement included perceptions of the app’s benefits and drawbacks, its relevance and quality, and the incorporation of behavior change techniques to promote health. User engagement is also influenced by individual health goals, user feedback, timely reminders, in-app social support, and coaching, with users having the potential to disengage and reengage as needed [82].

### Organization

Organizational barriers and facilitators play a significant role in the adoption and success of mobile apps in clinical care. Key barriers include staff resistance to change, limited resources, concerns about data privacy, interoperability challenges with existing systems, and the need for additional training [8]. Conversely, facilitators include strong leadership support, clear communication and education about benefits, seamless integration into workflows, evidence-based practice demonstrations, and user feedback engagement. Addressing these factors can optimize the adoption and integration of mobile apps into clinical care settings, promoting their effective use for improved patient outcomes.

### Wider System

Addressing system barriers requires a multifaceted approach involving collaboration among policy makers, health care providers, technology developers, and community stakeholders to create supportive regulatory frameworks, enhance infrastructure, improve digital literacy, and foster a culture of innovation and acceptance within the health care ecosystem. Key barriers to implementing and scaling health-related mobile apps and wearables include regulatory and legal challenges, particularly concerning data privacy, security, and compliance with medical device regulations across various regions. Additionally, interoperability issues with existing health care systems and electronic health records pose technical hurdles that demand complex solutions and coordination. Disparities in access to technology and digital literacy skills further contribute to a digital divide, limiting the effectiveness of mobile apps among underserved populations. Resistance from health care providers, along with insufficient training and support, hinders adoption, while low user engagement and adherence rates highlight the need to sustain motivation and address usability issues. Achieving sustainability and scalability requires careful resource allocation, long-term funding, and the development of viable business models to expand mobile app and wearable initiatives beyond the pilot and efficacy phases, effectively reaching broader populations.

## Social Media

### Condition

Social media broadly refers to the use of technology to connect individuals for communication and information sharing within virtual communities. The origins of social media, both as a concept and as a practice, can be traced to early internet systems with group communication features, such as bulletin board systems, USENET newsgroups, and online messaging services such as Internet Relay Chat. These platforms, popular in the 1980s, enabled communities of users with shared interests to communicate with one another. Early 1990s web-based distributed communities, such as webrings and GeoCities (GeoCities/Yahoo!), also represent early forms of social media. Webrings linked websites with similar content through embedded referential links, based on user self-identification and requests for inclusion to a “ringmaster.” GeoCities allowed users to define themselves through virtual geographic neighborhoods. However, the first platform explicitly designed to create and establish online social networks of family, friends, and acquaintances was Six Degrees (MacroView/YouthStream Media Networks), launched in 1997. Since then, global social media use has surged to over 5 billion users in 2024, representing 62.3% of the world’s population [83].

### Technology

Social media platforms are diverse, each focusing on different modalities for group-oriented communication. Content is published as posts, which may include text, images, audio, or video, depending on the platform. A key aspect of social media is the ability for users to interact with others’ posts. These interactions are typically permission-based and can be restricted to a closed-circle community or made available for public engagement, depending on platform settings and user preferences. Interaction types are as varied as content types and may include replies, reposts or shares with or without additional content, and acknowledgments of engagement such as likes, dislikes, and favorites. Examples of historical and current social media platforms include those focused on long-form essay and blog posting (eg, LiveJournal [Rambler Media Group], Blogger [Google LLC/Alphabet Inc.], Medium [A Medium Corporation]), short-post microblogging sites with posts originally limited to SMS text message length and remaining generally brief (eg, BlueSky [Bluesky Social, PBC], X [formerly Twitter], Mastodon [Mastodon gGmbH], Plurk [Plurk, Inc.]), image and video-oriented platforms (eg, Instagram [Meta Platforms], Pinterest [Pinterest, Inc.], YouTube [Google LLC], TikTok [ByteDance]), instant messaging platforms (eg, AOL Instant Messenger [AOL], Snapchat [Snap Inc.], QQ [Shenzhen Tencent Computer System Co., Ltd.], WhatsApp [Meta Platforms]), and multimodal social media sites (eg, Friendster [Friendster Labs Inc.], Myspace [Viant Technology LLC], Tumblr [Automattic], Baidu [Baidu, Inc.], Facebook [Meta Platforms]).

### Value Proposition

The primary value of social media for health communication, promotion, and intervention lies in its extensive reach, the ability to tailor messaging to specific community or user group interests at scale, and the speed of information transmission among platform members. Social media offers a relatively low-cost means of message promotion, with the potential for broad audience reach through reposting, thereby requiring minimal investment. The concept of “going viral,” where a social media post rapidly spreads from user to user, and even across platforms, at a speed and scope far beyond the initial audience, is often considered a measure of significant success in information dissemination. Another area of high value is the data analysis of social media content. Such analyses have been successfully used to identify emerging health concerns and health beliefs held at the population level among specific groups [84,85], trace outbreaks of foodborne illness [86], and track the spread of infectious diseases such as influenza and COVID-19 [87,88].

### Adopters

Individual adoption of social media use is so widespread as to be nearly ubiquitous among technology users. As of 2019, there were 3.5 billion people online, of whom 2.4 billion used Facebook, which at the time was the dominant social media platform. This means that 2 out of every 3 people online use social media [89]. Community formation on social media platforms may include individuals who know each other from offline environments or those who solely share similar interests and characteristics online. Trust and influence are also significant factors in social media adoption. Trust among the initial group of contacts who see an original post is extended across networks with each interaction through friend-of-a-friend social acceptance. Parasocial relationships with celebrities and influencers, established through the perception of interacting with their posts as if interacting directly with the person themselves, lead to additional credence being placed in social media content from such individuals. This, in turn, results in increased adoption of promoted messaging. However, social media adoption differs greatly by platform type and user demographic. Health promotion and outreach campaigns, patient recruitment initiatives, or community organizing involving social media can be extremely effective. However, they should consider the population distributions and primary languages of various platforms and tailor messages accordingly to achieve maximum reach and benefit [90].

### Organization

Engagement with social media platforms and systems for health communication at organizational levels can be influenced by institutional policies regarding who is authorized to make and respond to posts and what content is permissible for sharing with a public audience. Content experts who develop messaging and communications experts who tailor it for social media may be different individuals or teams. Often, the process of obtaining group or institutional approvals for specific content is much slower than the speed at which messages spread across social media. This delay can be problematic if required authorizations prevent an organization from responding effectively to questions or comments, or from addressing negative replies in a timely manner. In situations where a topic might be perceived as sensitive or controversial, a lack of response can be both detrimental and damaging. Understanding the cultural contexts of social media use in general, as well as the unique dynamics of specific platform environments, is essential for successfully developing and implementing health communication strategies on these networks.

### Wider System

In addition to the factors noted at the organizational level, health-related institutions at governmental levels often possess or are perceived to possess an authority that can make navigating questions of approachability and trust challenging. This is especially true in a social media environment where there must be a balance between adopting a less formal manner of communication and maintaining the formality required to accurately represent the organization and convey health-related content [91]. Differing policies among social media platforms can be challenging to navigate alongside health policies and regulations for data privacy and security, such as HIPAA. Important ethical considerations also arise, including how to maintain accountability and responsibility for content, combat misinformation, and respond to aggressive and harassing behaviors in social media spaces, whether directed at those who post health information or those who react to it.

### Embedding and Adaptation Over Time

In the last 25 years, social media has evolved from a niche technology to a central means of engaging communities and the public. Today, the majority of users under the age of 30 years consider social media sources to be as valid as, or even more valid than, traditional news media [92]. The challenge is that the same factors driving the positive aspects of social media can also lead to negative ones. For instance, the rapid pace of information distribution facilitates the spread of both misinformation and disinformation [93,94]. Social media interventions have proven successful in improving mental health status through interpersonal connections and increasing self-esteem and belonging. However, social media can also promote poor self-image through comparisons with artificially high standards, serve as a source of extreme stress, and potentially worsen mental health status [95]. Future work in using social media for health communication and intervention must be mindful of these considerations to avoid unintended consequences.

## Discussion

By applying the NASSS framework, this paper elucidates the complex interplay among individual, organizational, and societal factors influencing the adoption and implementation of mHealth technologies. The framework provides a comprehensive perspective to understand the dynamics of technology adoption in health care over the last 25 years. Each mobile technology offers distinct advantages and disadvantages for promoting public health and health care services. SMS text messaging is widely accessible and effective for reaching large populations, especially in low-resource settings. Its simplicity has proven successful in behavior change and medication adherence. However, SMS text messaging is limited in functionality, supporting only basic text communication, which may be less engaging for users seeking interactive or multimedia experiences. Mobile apps and wearables offer a comprehensive range of functionalities, including health tracking, personalized coaching, and integration with wearables, making them ideal for in-depth health management. Yet, they face higher adoption barriers due to the need for smartphones, app literacy, and potential costs, with user engagement often declining over time. Social media platforms, with their extensive reach and rapid information dissemination capabilities, are valuable for building communities and promoting health interventions. However, they carry risks such as the spread of misinformation and require careful management of privacy and data security. Each mobile technology serves different user needs and contexts, highlighting the importance of choosing the appropriate tool for specific health interventions.

Our retrospective discussion highlights the potential of SMS text messaging, mobile apps, wearables, and social media to promote healthy behavior and manage diverse health conditions, underscored by their accessibility and ease of implementation. However, challenges such as provider reluctance, accessibility barriers, and regulatory constraints impede the widespread adoption and integration of mHealth into health care delivery systems. SMS text messaging, while effective, often leads to abandonment due to its limited interactivity, as users may seek more dynamic tools. Mobile apps, despite their comprehensive features, face adoption challenges, including the need for smartphones, app literacy, and potential costs, with user engagement often declining if the apps are not well-designed or integrated into clinical or daily workflows. Social media campaigns struggle with user engagement due to misinformation risks, privacy concerns, and the need for constant content management, which can lead to distrust and abandonment.

Moreover, we emphasize the importance of addressing sociocultural variability and literacy concerns to enhance the inclusivity and effectiveness of mHealth interventions. While these technologies offer personalized resources and empower users to actively participate in their health care, challenges such as limited scientific evidence, technological complexity, and organizational barriers hinder their widespread adoption and impact. Moreover, disparities in access, digital literacy, and user engagement underscore the need for a holistic approach to address systemic barriers and optimize the effectiveness of mHealth interventions.

The last 25 years have seen remarkable strides in adopting and adapting mHealth technologies, driven by technological innovation and a growing recognition of their potential to revolutionize health care delivery. Nevertheless, continued efforts are needed to address persistent challenges and mitigate instances of abandonment, ensuring that mHealth interventions can fully realize their potential in improving health outcomes and advancing equitable access to care.

## Abbreviations

AI: artificial intelligence
API: application programming interface
HIPAA: Health Insurance Portability and Accountability Act
mHealth: mobile health
NASSS: Nonadoption, Abandonment, Scale-Up, Spread, and Sustainability
